# Methylation changes in the TFAP2E promoter region are associated with BRAF mutation and poorer overall & disease free survival in colorectal cancer

**DOI:** 10.18632/oncoscience.149

**Published:** 2015-03-23

**Authors:** Andrew D. Beggs, Mark P. Dilworth, Enric Domingo, Rachel Midgley, David Kerr, Ian P.M. Tomlinson, Gary W. Middleton

**Affiliations:** ^1^ Translational Surgical Biology Laboratory, School of Cancer Sciences, University of Birmingham, Birmingham, UK; ^2^ Molecular & Population Genetics Laboratory, Wellcome Trust Centre for Human Genetics, University of Oxford, Oxford, UK; ^3^ Department of Oncology, University of Oxford, Oxford, UK; ^4^ School of Cancer Sciences, University of Birmingham, Birmingham, UK

**Keywords:** BRAF mutation, chemoresistance, TFAP2E, Wnt signalling

## Abstract

**Introduction:**

BRAF mutant colorectal cancer carries a poor prognosis which is thought to be related to poor response to conventional chemotherapy. BRAF mutation is associated with the serrated tumour phenotype. We hypothesised that one of the mechanisms by which BRAF mutant colorectal cancer demonstrate poor outcomes with chemotherapy is abnormal gene methylation

**Methods:**

The Cancer Genome Atlas (TCGA) methylation data was analysed using a linear regression model with BRAF mutation as an independent variable. Expression datasets were also obtained to correlate functional changes. Top differentially methylated probes were taken forward for validation by methylation pyrosequencing. These probes were analysed on a cohort of patients enriched for BRAF mutations taken from the VICTOR and QUASAR2 studies.

**Results:**

In an analysis of 91 tumours (9 BRAF mutant, 82 wild type), the Illumina probe cg11835197 was the probe identified as top differentially methylated (p = 2.56×10-7, Bayes Factor (BF) =6.54). This probe covered a region −413bp from the promoter region of TFAP2E. We found a complex pattern of CpG specific methylation of this region which was associated with both overall (p=0.044) and disease free (p=0.046) survival.

**Discussion:**

BRAF mutant tumours may attain part of their chemoresistance from abnormal TFAP2E methylation, which has not previously been described.

## INTRODUCTION

Previous studies have clearly shown that patients with BRAF mutant colorectal cancer (CRC) receiving adjuvant chemotherapy following resection have a worse outcome compared to their BRAF wild type counterparts [1, 2]. This inferior outcome appears to specifically relate to patients receiving adjuvant 5-fluorouracil/leucovorin (FU/LV) alone. In the CALGB 89803 study BRAF mutant stage III patients treated on the control arm of FU/LV alone had a significantly reduced disease free survival (DFS) (HR = 1.83) and overall survival (OS) (HR = 2.43) compared with BRAF wild type patients treated with the same regimen [1]. This inferior outcome was not seen in BRAF mutant patients treated with IFL and indeed there was a trend towards improved outcome for the addition of Irinotecan in BRAF mutant patients, a trend that was not seen in BRAF wild type patients. In a combined analysis of the National Surgical Adjuvant Breast and Bowel Project C-07 and C-08 trials of adjuvant therapy OS was again inferior in BRAF mutant microsatellite stable (MSS) patients: BRAF mutant patients with deficient mismatch repair (MSI) had outcomes similar to BRAF wt MSS patients [2]. In the C-07 study which demonstrated a benefit for the addition of oxaliplatin to adjuvant FU/LV, there was no significant interaction between BRAF mutation status and the beneficial effect of oxaliplatin.

The molecular basis of this inferior outcome of BRAF mutant patients receiving adjuvant FU/LV is unknown. A recent study has demonstrated an important link between hypermethylation and chemo-resistance in CRC [3]. Fifty-one percent of CRC patient samples were found to have hypermethylation of TFAP2E. TFAP2E is a member of the AP2 family of transcription factors 6 and has a putative link as a tumour suppressor. The AP2 transcription factor family consist of five subtypes, AP2- α,β,χ,δ and AP2-ε, and are located predominantly in the nucleus where they regulate transcription and interact with other signal transduction pathways., AP2-a has been shown to modulate the Wnt signalling pathway 7 by interacting with the Adenomatous Polyposis Coli (APC) protein, the key protein in colorectal cancer development. It was shown that TFAP2E negatively regulated DKK4 and expression of DKK4 mediated chemoresistance to fluorouracil but not to irinotecan or oxaliplatin. In a cohort of patients treated with fluorouracil/oxaliplatin TFAP2E methylation was significantly associated with lack of response to therapy. The authors hypothesised that TFAP2E hypermethylation mediated clinical resistance to fluoropyrimidine based doublet therapy via DKK4- mediated fluorouracil resistance.

Zhang et al [4] used high resolution melt analysis of TFAP2E methylation in 311 colorectal cancer patients. They found that hypermethylation conferred a survival advantage in these patients, and that patients with hypermethylation in TFAP2E presented with earlier stage tumours, had less invasion, fewer positive lymph nodes and had better tumour differentiation.

A specific association with TFAP2E hypermethylation and BRAF mutation which in all stages is associated with the hypermethylator phenotype was not made in either of these studies. It has recently been described [5] that transcriptional control of gene expression via promoter methylation is a more complex process that previously understood. In fact, hypomethylation within CpG shore regions as well as hypermethylation, can cause decreased expression of a gene. Vanderkraats et al [5] demonstrated patterns of adjacent short stretches of hypermethylation followed by hypomethylation downstream of the transcription start site of a gene were the most strongly linked correlates with reduction of expression of genes.

Given the well documented association between BRAF mutant CRC and the CpG island methylator phenotype (CIMP) [6, 7], we examined the colorectal cancer TCGA to identify genes that were highly differentially methylated between BRAF mutant and wild type cases to identify candidates worthy of further analysis to unravel the clinical finding of poor outcomes after adjuvant FU/LV. We found that the most significant hit was a probe associated with the TFAP2E gene. This was intriguing given the data of Ebert and colleagues who had shown that hypermethylation of TFAP2E was a marker of FU resistance (not irinotecan or oxaliplatin resistance) in colorectal cancer. However, in that study there was no reported differential methylation in TFAP2E between BRAF mutant and wild type CRC. This prompted us to undertake a precise evaluation of methylation across TFAP2E in BRAF mutant CRC in order to understand its full complexity

## RESULTS

### Initial discovery phase

**Figure 1:**
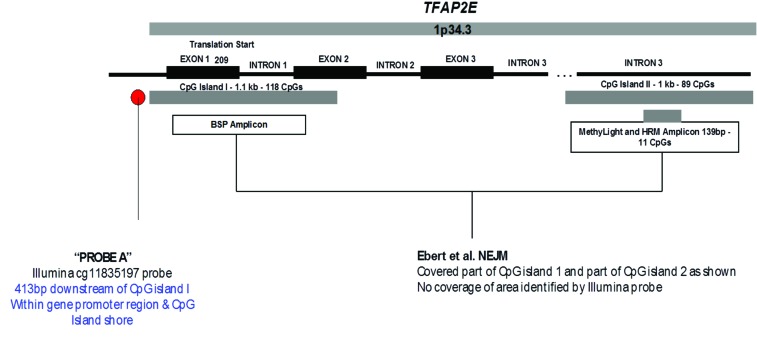
Diagrammatic representation of TFAP2E CpG islands and their relationship to the TFAP2E gene (Adapted from Ebert et al)

For the TCGA methylation dataset, 92 level 1 methylation dataset files (as at the time of the study, these were the only ones available for level 1 download along with mutation data) were retrieved for tumour samples, of which 9 possessed BRAF V600E mutations, and 83 were wild type for BRAF mutations. Analysis was successful and the top hit was Illumina probe cg11835197 (Chr1:36038515-36038515) with a log fold change of 1.73, p = 2.56×10-7, Bayes Factor (BF) =6.54. This was just upstream of the 1^st^ CpG island of TFAP2E (Figure 1, Probe A), the probe being −413bp of the island start site. TFAP2E is a gene involved as a upstream regulator of DKK4, a Wnt signalling pathway associated gene, previously described as being implicated in chemoresistance to 5-FU chemotherapy by Ebert et al[3]. The remaining 6 probes, all with BF > 5 are shown in Table 1. The genes identified in this set include *GDPD2* (which hydrolyses glycerophosphoinositol to produce inositol 1-phosphate and glycerol), *SETX* (a RNA helicase), ACSL5 (a bHLH transcription factor), *EPOR* (the erythropoietin receptor), PEG10 (paternally expressed 10, a gene of unknown function), and a CpG island adjacent to *ZC3H3* and *RHPN1*.

**Table 1:** Table of top ranked probe identifiers ranked by Bayes factor (largest first) comparing BRAF mutant vs. BRAF wild type tumours in TCGA methylation dataset

Rank	Illumina probe ID	Log [Fold change]	t	P Value	Adjusted p value	Bayes factor	Gene
1	cg11835197	1.73	5.55	2.56E-07	0.003	6.54	*TFAP2E*
2	cg25685838	2.17	5.41	4.77E-07	0.003	5.97	*GDPD2*
3	cg06353948	3.19	5.37	5.61E-07	0.003	5.83	*SETX*
4	cg13849691	3.35	5.37	5.66E-07	0.003	5.82	*ACSL5*
5	cg24477567	1.99	5.20	1.15E-06	0.003	5.18	*EPOR*
6	cg06695761	1.63	5.18	1.23E-06	0.003	5.13	*PEG10*
7	cg03870862	2.20	5.18	1.25E-06	0.003	5.11	*ZC3H3*

On further study of the Ebert et al paper, it was noted that TFAP2E possesses two associated CpG islands, one within exon 1 (CpG Island I) of TFAP2E and other within intron 3 (CpG Island II, Figure 1). In the Ebert paper, the island (CpG Island II) within intron 3 was noted to be differentially hypermethylated in chemoresistant tumours; however they found that that the island within exon 1 was universally hypermethylated by bisulphite sequencing. Probe A identified by our study lies at 413bp from the start of CpG island 1, within the shore of the promoter associated CpG island of TFAP2E. This region had not specifically been studied in detail by Ebert et al as it was thought to be uniformly hypermethylated. Correlation of TFAP2E methylation at cg11835197 with expression was obtained using the TCGA dataset, and demonstrated that there was a weak but significant negative correlation between TFAP2E methylation and expression at our identified region (Spearman p=0.044, rho = −0.13), i.e. as methylation of TFAP2E in this region increased, expression of TFAP2E decreased. We checked methylation in the Ebert region against TFAP2E gene expression, and also found significant correlations (Spearman p=0.04).

Because of our findings and because differential methylation observed within the shores of CpG islands are the most transcriptionally relevant[5], we decided to study this region, and the original region described by Ebert et al in more detail on an enriched BRAF mutant study set.

### Validation

We successfully carried out bisulphite pyrosequencing on all supplied samples from the VICTOR and QUASAR2 studies. Of the 96 supplied tumour samples, 83 were BRAF mutant (all V600E) as previously determined by Sanger sequencing. A simple analysis, comparing BRAF mutation status vs. methylation was carried out. At the area downstream of CpG island 1 (probe cg11835197), which contained 2 CpG's, the first CpG, corresponding to Illumina probe ID cg11835197 seen in the array experiment was not significantly differentially hypermethylated (p=0.15, Wilcoxon). As a consequence of the pyrosequencing assay design, the assay covered a second CpG +6bp upstream of cg11835197, which on analysis was found to be significantly differentially hypomethylated (p=0.0032, Wilcoxon), with BRAF mutant tumours having a median of 18.5% for mutated tumours and 26.0% for wild type tumours.

Methylation of DNA is a complex phenomenon and may be affected the factors such as age (which increases methylation linearly), gender (bias towards females have hypermethylated tumours) and co-incident methylation of adjacent CpG's. Because of the potential for bias by these factors, we constructed a multivariate logistic regression model to correct for the effects of these potential confounders. We used BRAF mutation status as the dependent variable and percentage methylation in our identified region at CpG1 and CpG2, MSI status, age, gender, and CIN status. Variables were removed sequentially from the model at a threshold of p<0.05, using reverse stepwise methodology. In this model (table 2), the methylation of TFAP2E was shown to increase significantly in CpG1 (coef = 0.260, z=2.16, p=0.022), and significantly decrease in CpG2 (coef =−0.258, z=−2.55, p=0.008) in BRAF mutated tumours.

**Table 2:** Adjusted logistic regression model of BRAF mutation status vs. methylation at region surrounding Illumina probe ID cg11835197 consisting of 2 CpG's

Variable	Coefficient	95% CI	p-value
CpG 1	0.260	0.038-0.482	0.022
CpG 2	−0.258	−0.448--0.068	0.008
Chromosomally unstable tumour (1=CIN)	−1.266	−2.905-0.373	0.13
Gender (1=male)	−1.535	−3.149-0.078	0.062
MSI tumour (1= MSI)	0.486	−1.379-2.352	0.609
Age	0.056	−0.032-0.145	0.214

We also carried out “safety checks” on this model to ensure bias was not being introduced inadvertently. BRAF mutation is known to be associated with tumours occurring in females and we found that in this cohort was shown to be negatively correlated with male gender (coef = −1.54, z=−2.16, p=0.062). Chromosomal instability and BRAF mutation are also almost always mutually exclusive and in this cohort BRAF mutation and chromosomal instability tended towards being mutually exclusive (coef = −1.27, z=−2.23, p=0.13). A Hoesmer-Lemeshow goodness of fit test demonstrated a good model fit for the dataset (p=0.36).

We then decided to compare our results to the previously observed region of differential hypermethylation in CpG Island II from Ebert et al. In CpG island II, which contained 11 CpG's, when added to our logistic regression model, none attained significance associated with BRAF mutation (Table 3), setting the threshold at p<0.05. We observed uniform hypermethylation across the Ebert region, with no differential methylation seen between BRAF mutant and BRAF wild type tumours.

**Table 3:** Table of percentage methylation across differentially methylated region identified by Ebert et al within intronic region of TFAP2E

	Average methylation		
CpG	BRAF Wild type	BRAF mutant	p-value (Wilcoxon)
1	38.7%	45.5%	0.18
2	40.5%	47.0%	0.21
3	36.8%	44.5%	0.14
4	34.5%	41.2%	0.12
5	34.3%	41.5%	0.15
6	36.2%	42.0%	0.27
7	40.1%	48.5%	0.12
8	45.6%	55.6%	0.11
9	36.7%	43.7%	0.17
10	33.3%	40.7%	0.12
11	27.7%	34.8%	0.14

**Table 4:** Table of cox regression model of TFAP2E methylation in overall and disease free survival

Variable		Overall survival			Disease free survival		
		HR	95% CI	P	HR	95% CI	P
TFAP2E CpG1	Above thresholdBelow threshold (baseline)	1.08 -	0.43-2.83 -	0.844 -	0.90 -	0.37-2.17 -	0.818 -
TFAP2E CpG2	Above thresholdBelow threshold (baseline)	0.34 -	0.12-0.97 -	0.044 -	0.40 -	0.16-0.98 -	0.046 -
MSI status	Microsatellite unstableMicrosatellite stable (baseline)	0.45 -	0.172-1.18 -	0.104 -	0.34 -	0.14-0.84 -	0.019 -
Gender	MaleFemale (baseline)	1.26 -	0.55-2.93 -	0.581 -	1.45 -	0.69-3.08 -	0.330 -
Age (years)		1.04	0.98-1.09	0.151	1.03	0.98-1.08	0.200
Chemotherapy	Chemotherapy given Chemotherapy not given (base- line)	2.45 -	0.52-11.46 -	0.255 -	2.57 -	0.66-10.1 -	0.175 -
Location	Left colonRight colon (baseline)	1.13 -	0.42-3.06 -	0.811 -	1.14 -	0.47-2.74 -	0.769 -
KRAS mutation	MutationNo mutation (baseline)	9.61 -	0.79-116.6 -	0.076 -	9.27 -	1.42 -	60.7 -
Stage	T4< T4 (baseline)	0.62 -	0.26-1.47 -	0.278 -	0.73 -	0.33-1.61 -	0.435 -
Lymph node status(1=LN positive)	Positive lymph nodesNegative lymph nodes (baseline)	1.73 -	0.62-4.87 -	0.298 -	2.57 -	0.65-10.08 -	0.175 -

### Effect on survival in the VICTOR & QUASAR2 cohorts

In order to model the effect on survival on the VICTOR & QUASAR2 cohort for whom methylation data and survival data was available (n=96), we constructed a multivariate Cox regression model comparing overall survival (OS) and disease free survival with available clinicopathological variables. These variables were age, gender (male = 1, female = 0), use of chemotherapy, chromosomal instability, microsatellite instability status, KRAS mutation status and BRAF mutation status (in order to correct for the confounder of poorer survival in BRAF mutant patient). In order to more easily model the effect of changes in methylation at the 1^st^ and 2^nd^ CpG's at Probe A in TFAP2E, we thresholded the variable such that a cut-off of 20% was chosen to differentiate between the “hypermethylated” and “hypomethylated” groups, based on a non-parametric receiver-operator curve analysis demonstrating that this percentage methylation differentiated maximally between the two groups of methylation.

**Figure 2:**
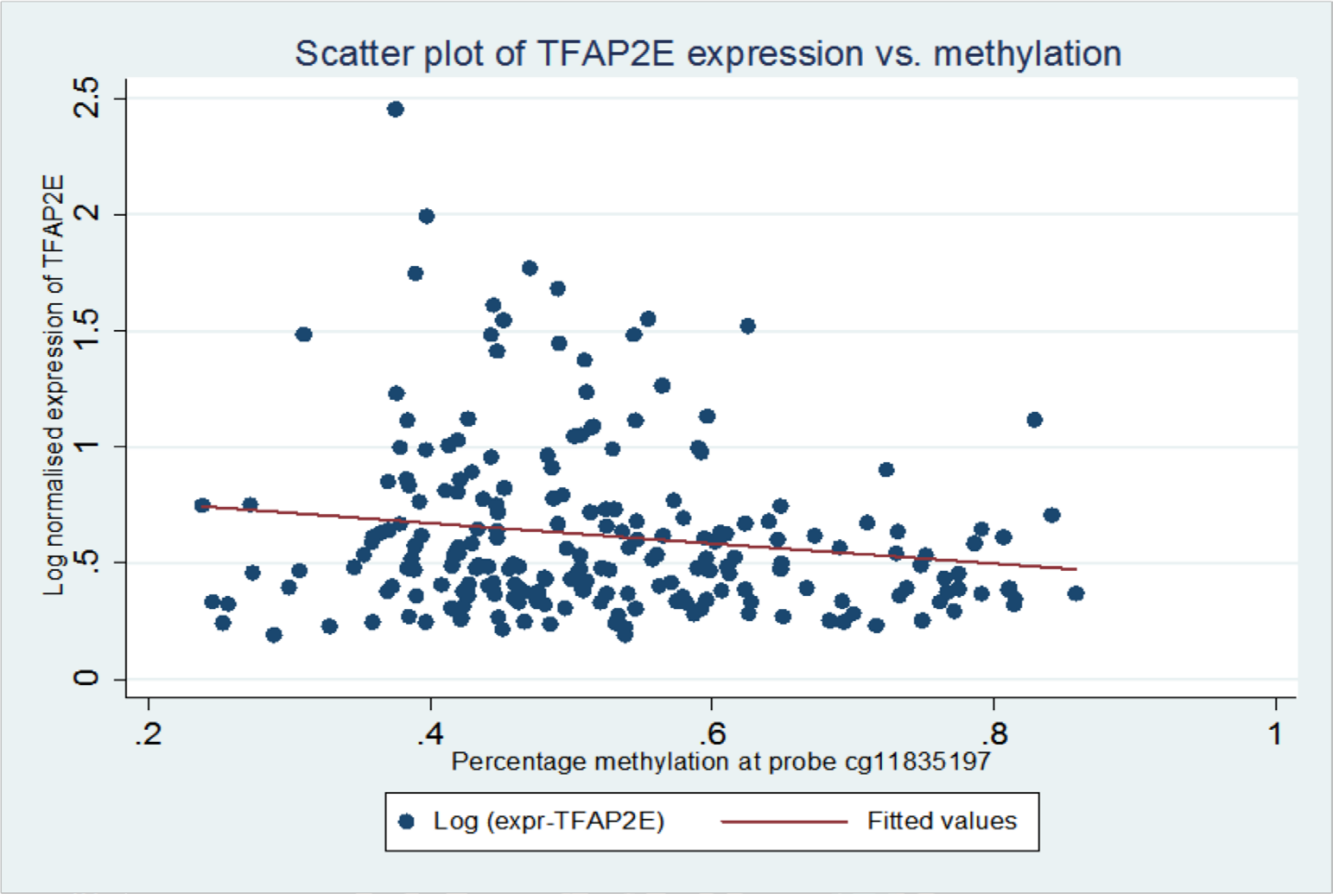
Scatter plot of TFAP2E expression vs methylation showing correlation between methylation of TFAP2E expression and methylation at probe cg11835197.

**Figure 3:**
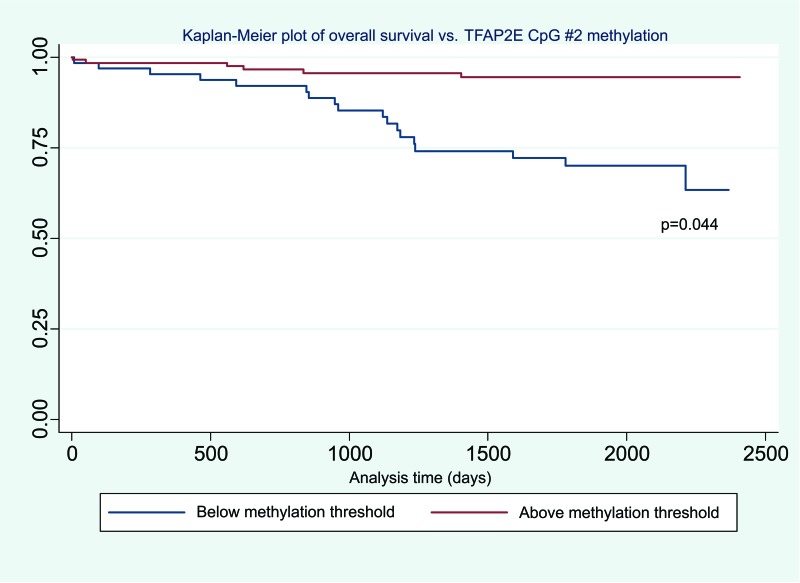
Kaplan-Meier plot of overall survival vs TFAP2E methylation showing decreased overall survival in hypomethylated tumours.

Using this cut-off, the relationship between TFAP2E hypermethylation at CpG1 and both overall (p=0.844) and disease free survival (p=0.818) did not reach significance. For hypomethylation at CpG2, there were significant associations between both overall (p=0.044) and disease- free (p=0.046) survival (Figure 2 & Figure 3). We did not find any significant association between BRAF mutation and either overall or disease free survival when corrected for overall TFAP2E methylation.

**Figure 4:**
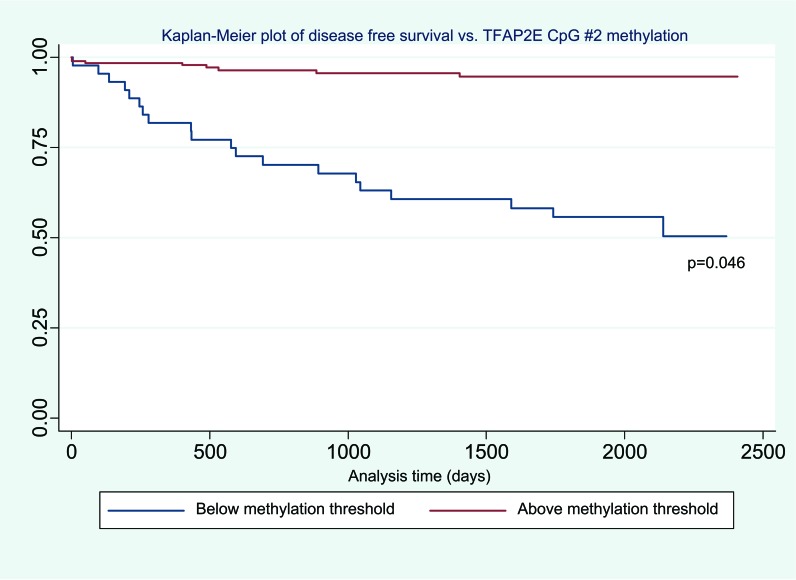
Kaplan-Meier plot of disease free survival vs TFAP2E methylation showing decreased disease free survival in hypomethylated tumours

On examination of CpG island II in the intronic region, because of the uniformity of methylation observed, we took the average methylation across all 11 CpG's and used it in a Cox regression model, correcting for age, gender, chemotherapy status, MSI status, CIN status and BRAF/KRAS mutation status. There was no association between methylation across CpG island II and overall (HR 1.38, 95% CI 0.16-12.1, p=0.771) or disease free (HR 1.76, 95% CI 0.21-14.75, p=0.602) survival.

## CONCLUSIONS

In our study we have used the TCGA dataset to identify the changes that are seen in methylation in BRAF mutant tumours. We found, using an unbiased hypothesis free approach that these tumours are associated with changes in methylation across the TFAP2E promoter region, specifically locus specific hypomethylation of a CpG within the shore of the CpG island of TFAP2E associated promoter region. Ebert et al [3] examined the rates of BRAF mutation in association with promoter hypermethylation in their study group, however they found that there was no association with BRAF mutation. This is because the region of the CpG island they studied was not the one that was transcriptionally relevant to TFAP2E expression, and the use of Methylight missed subtle single CpG changes that are important in the regulation of transcription. We believe this as their observed region had little variation in methylation observed using pyrosequencing; thus the differences observed in expression could not be related to methylation change at this region, despite the fact we have also demonstrated an association between methylation and expression here. The observed phenomenon whereby BRAF mutated tumours demonstrate resistance to 5-FU-based chemotherapy may be associated with the phenomenon of differential changes in TFAP2E promoter methylation.

We found similar outcomes to Zhang et al [4], in that hypomethylation in TFAP2E was associated with poorer survival. However, the location of hypomethylation was different in our study, with hypomethylation in the CpG shore downstream of the promoter region being significantly associated with prognosis. In the Zhang paper, they found hypomethylation within the region described by Ebert et al [3] within an intronic region of TFAP2E. They used a high resolution methylation melt analysis which covered 11 CpG's. This has the weakness in that all CpG's are treated as a single unit [8], rather than in pyrosequencing which was utilised in our study which can detect subtle single CpG changes in methylation.

We agree that TFAP2E expression is linked to chemoresistance, however we believe the mechanism of this resistance via promoter methylation is more complex. Our study has demonstrated that there is a significant association on multivariate analysis towards hypermethylation in the 1^st^ CpG and hypomethylation in the 2^nd^ CpG of our identified region, and this is linked to both BRAF mutation status and lower overall and disease free survival. This would fit with the fine control of gene expression seen by Vanderkraats et al [5], whereby regions of hypermethylation followed by hypomethylation is seen, and transcriptional control to the single CpG level has been demonstrated in multiple studies [9, 10]. Using the TCGA dataset, we have also demonstrated that methylation change in this region is linked to TFAP2E expression.

It was found by Ebert et al [3] that cell lines transfected with a TFAP2E clone (leading to overexpression) had poorer survival, i.e. they were chemosensitive, however they did not find a conclusive link between methylation in their CpG island and TFAP2E expression. Ebert et al [3] used 5-azacytidine to demonstrate changes in TFAP2E expression in cell lines, however they did not find conclusive changes in all cell lines studied, with only 2 cell lines demonstrating a change in TFAP2E expression with 5-azacytidine exposure. The use of 5-azacytidine, which causes global hypomethylation across the genome is the only tool currently available to study methylation changes in cells and because of its genome wide effects may lead to the loss of subtle mechanistic changes.

We have not replicated the finding by Ebert et al [3] that hypermethylation in their identified region is associated with poorer outcome on adjuvant FU/LV. We have found, that hypomethylation within a specific CpG identified in our study is related to both overall and disease free survival following adjuvant FU/LV based therapy; however this may be a phenomenon related to the fact that these tumours are BRAF mutated and thus will have a poorer survival, however we took account of this in our regression model and TFAP2E methylation change still remained significant. We agree that TFAP2E/DKK4 plays a role in chemoresistance, and we further propose that this is also linked with BRAF mutation and is potentially also responsible for their chemoresistance via dysregulation of the MAPK pathway. Further study is needed in a larger cohort to identify the potential of these identified changes, and also to fully understand the mechanisms that lead to BRAF tumours becoming chemoresistant and having a poorer prognosis, as this is unlikely to be due to a single pathway phenomenon.

## MATERIALS AND METHODS

### Discovery phase using TCGA dataset

In order to carry out an initial analysis of differences in hypermethylation between *BRAF* mutant and *BRAF* wild type tumours, Level 1 Illumina Human Methylation

450 data from the COAD colon cancer dataset from the NIH Cancer Genome Atlas (TCGA) was downloaded from the data repository in August 2013 (https://tcga-data.nci.nih.gov/tcga/). This data was correlated by BRAF mutation status by downloading mutation data from sequencing datasets from the same repository. Sample IDs were correlated using the TCGA sample ID.

Level 1 methylation data was imported in R v 2.6.1, filtered according to the methodology of Triche et al [11], SWAN normalised [12] and an linear fit model with empirical shrinkage of T-statistics fitted, using *BRAF* mutation status as a dependent variable, correcting for age and gender. Multiple testing correction was performed using the Benjamini-Hochberg (BH) adjustment. Top probes were sorted by Bayes factor (with significant BF > 5) and exported to Microsoft Excel for correlation with Illumina probe identifiers as well as chromosomal coordinates.

### Assay design & validation on colorectal tumour set

In order to study a population enriched for BRAF mutation, 96 tumour DNA samples from the VICTOR and QUASAR2 trials were used. The VICTOR study [13] was a randomised controlled trial of rofecoxib, a COX2 inhibitor against placebo in 908 patients in the post-adjuvant prevention of recurrence of colorectal cancer. The QUASAR2 study [14] was a randomised controlled trial comparing capecitabine vs. capecitabine + bevacizumab as adjuvant therapy for CRC. The VICTOR study received ethical approval from West Midlands Multicentre Research Ethics Committee and the QUASAR2 study from the Metropolitan Multi-centre Research Ethics Committee (ref: 04/MRE11/18). DNA extracted from the VICTOR and QUASAR2 samples was enriched for tumour material by macrodissection from slides. Samples were also previously genotyped for KRAS, NRAS, PIK3CA, TP53 and CDC4 mutations by Sanger sequencing, chromosomal instability status using image cytometry ploidy [15] and microsatellite instability status by genotyping of the BAT25, BAT26 and D2S123 polymorphic loci.

Top hits from the methylation association study were validated by means of bisulphite pyrosequencing. Assay design was carried out using Qiagen Pyromark 2.0 software. The region of interest was chosen flanking the Illumina probe sequencing +/−200bp, with the sequence being exported from the UCSC Genome Browser (Genome v37 release). Standard assay design conditions were utilised, with the target CpG from the probe highlighted as the region of interest.

Two sets of probes designed for study of *TFAP2E* methylation. For study of the differentially hypermethylated site observed in the TCGA dataset (TFAP2E-Probeset-1), primers designed were Fwd- AGTAGATAGGTTGGAGTTTTTAGTTTATA; Rev – [Btn] CCTTACCTTTAAACAAAACACTATTCT; Seq – GGTTGGAGTTTTTAGTTTATAA. The designed amplicon encompassed two CpG's – firstly the CpG covered by the Illumina probe and the second +6bp upstream of the first probe. For study of the original TFAP2E hypermethylation site seen by Ebert et al, primers designed were Fwd – TTGGTGAGAAAGGGAGGTAGTT; Rev - [Btn] ACCCTACCAACTCCAAATACCTCTAC and Seq - TGTAGTTTTAGTTTTATTTTAGAAG and covered eleven CpG's within this region.

Primers were utilised in standard (20uM) concentrations in a PCR reaction using the Pyromark PCR kit. For each PCR reaction, 1uL of bisulphite converted DNA was made up in a reaction with 12.5uL of Pyromark PCR master mix, 0.3uL of forward primer, 0.3uL of reverse biotinylated primer, 3uL of Q solution, 5uL of Coral Load concentrate, 1.5uL of 20mM MgCl made up to a total of 25uL reaction volume with ddH O. PCR conditions were according to Qiagen specifications with an annealing temperature of 56C (experimentally determined by gradient PCR). After PCR, 5uL of product was run on a 1.5% agarose gel to ascertain success of the reaction and successful reactions were taken forward to pyrosequencing. Pyrosequencing was performed according to manufacturer's specifications using a Qiagen PyroMark 96 ID instrument, diluting 20uM sequencing primer to 1:50 for use in sequencing. Pyrosequencing runs were subjected to quality control using Qiagen Pyro Q-CPG software and only reactions passing QC measures were used. A random selection of 10% of runs was duplicated to ensure consistency.

### Expression/methylation correlation

In order to validate the correlation between expression and methylation in TFAP2E, Level 3 data for gene expression determined via RNA-seq and methylation data via the Illumina HumanMethylation450 BeadChip was downloaded from the TCGA data portal in November 2013 (https://tcga-data.nci.nih.gov/tcga/). Expression data was log-normalised and correlated with methylation via Spearman's' Rho using Stata 12.1 (StataCORP, TX)

### Outcomes for TFAP2E on VICTOR/QUASAR2

Survival data was available for both the VICTOR study and QUASAR2 studies, in this a multivariate Cox regression model was set up using overall and disease free survival as separate dependent variables with mutational status, microsatellite instability status, age, T stage, N stage, chemotherapy status and location (right colon vs. left colon). All statistical analysis was performed in Stata 12.1 (StataCORP, TX).
